# Special Issue on “Clinical Research of Spontaneous Pneumothorax”

**DOI:** 10.3390/jcm11112988

**Published:** 2022-05-25

**Authors:** Paola Ciriaco

**Affiliations:** Department of Thoracic Surgery, Scientific Institute and University Vita-Salute San Raffaele, Hospital San Raffaele Milano, 20132 Milan, Italy; ciriaco.paola@hsr.it

Spontaneous pneumothorax (SP) may occur in the apparent absence of disease (primary spontaneous pneumothorax PSP) or as a consequence of an underlying condition (secondary spontaneous pneumothorax SSP). Relatively little attention has been dedicated to this condition in terms of pathophysiology and systematic management.

This Special Issue in the *Journal of Clinical Medicine (JCM)* brings together a series of high-quality scientific contributions that try to weave a common thread that extends from the etiology to the treatment of SP [1,2,3,4,5]. The covered topics distinctly point out the issues related to PSP and SSP, although both diseases present some similar approaches in the diagnostic–therapeutic path.

The review by Halifax R [1] reports that PSP is more common in ectomorphic patients (tall and thin), with a low body mass index, and among smokers. A PSP is considered to occur without apparent cause and in the absence of significant lung disease. One of the important issues to emerge from this review is that the lungs of these patients are rarely “normal”. They might present with blebs or bullae that are often intact, contrary to the historical postulate that it is the rupture of these that causes pneumothorax. In some cases, no macroscopic lesion is seen at all during surgery [6], supporting the thesis that air might also leak from the thinned visceral pleura.

Management of SP over the decades has been mainly conservative and, until now, no randomized trials have compared conservative treatment and surgery [7]. The indications for treatment were provided by the American College of Chest Physicians in 2001 and the European Respiratory Society in 2015 [8,9]. The European Respiratory Society reached the consensus on “Indications for definitive management of primary spontaneous pneumothorax” that should be offered to those with a second episode of PSP, persisting air leak of >3–5 days, hemopneumothorax, bilateral pneumothorax and profession at risk [2,9]. Still debated is the possibility of offering video-assisted thoracoscopic surgery (VATS) for every first episode of PSP [2]. Available studies have attempted to identify a subgroup of PSP patients that could benefit from surgery during their first episode. Only two randomized controlled trials showed the superiority of surgery in terms of ipsilateral recurrence, and in those patients whose high-resolution computed tomography demonstrated bullae ≥2 cm [10]. Nevertheless, it is not clear if a SP occurring in patients with bullae ≥2 cm should be considered primitive without underlying pulmonary disease [1].

VATS has reached established popularity among thoracic surgeons offering the choice of definitive surgical treatment of SP with decreased pain, length of hospital stay, and morbidity. Nowadays, VATS is the preferred surgical approach over open thoracotomy, due to less invasiveness and comparable results in terms of success rate. VATS is classically performed using two or three ports. Experience with uniportal VATS is increasing and it seems to be associated with reduced patient postoperative pain and reduced paresthesia, which is surely appreciated by patients [11]. Unfortunately, this technique requires greater skill to manage surgical instruments in smaller, less accessible areas [12] and it is performed in few centers.

The target of SP treatment is the resolution of symptoms and prevention of recurrences. Surgery for PSP consists of the resection of visible blebs and/or bullae, which occur in almost 80% of cases, and it is generally completed with a pleurodesis procedure [2,13]. Pleurodesis can be achieved mechanically (partial parietal pleurectomy, abrasion, electrocauterization) or chemically (talc insufflation or instillation of chemical agent) [2]. Chemical pleurodesis with talc insufflation seems to offer greater protection against pneumothorax relapse; still, the advisability of administering talc in young patients remains debated [14,15].

SSP, as defined by the British Thoracic Society (BTS) guidelines [16], is a pneumothorax occurring in patients with underlying lung disease, or in patients over 50 years of age with a significant smoking history. Underlying lung diseases include chronic obstructive pulmonary disease, interstitial lung disease, lung infections (i.e., pneumocystis, COVID-19, tuberculosis), lung cancer, and endometriosis [3,5]. SSP is more common than PSP, representing, according to English SP epidemiological data, over 60% of pneumothoraces. Its outcome is worse than PSP, with a higher recurrence rate and longer hospitalization [17]. The BTS guidelines, in absence of hemodynamic instability, recommend initial conservative treatment with a pneumothorax at the hilum <1 cm. Needle aspiration and chest tube drainage, along with hospitalization, are considered for pneumothorax at the hilum 1–2 cm or >2 cm, respectively [16]. Choice of surgery presents some differences compared to PSP. Multidisciplinary discussion is advocated in case of air leak persistence, considering the higher rate of thoracotomy in this category [18].

Catamenial pneumothorax (CP), on the other hand, deserves a separate discussion. CP is defined as recurrent spontaneous pneumothorax occurring in fertile women between 24 h before and 72 h after the onset of menstruation. It has always been considered an unusual condition [19], but since its recognition has improved, its frequency is nowadays quoted between 23 to 30% of pneumothoraxes among women [20]. CP is considered one of the most frequent expressions of the Thoracic Endometriosis Syndrome (TES) [21,22], consisting of the presence of endometriotic lesions in the lungs and pleura and comprising four clinical entities, such as catamenial pneumothorax, catamenial hemothorax, catamenial hemoptysis, or lung nodules [23].

Since TES and pelvic endometriosis can be associated in 60% of cases [24], management of CP should be carried out by a multidisciplinary team that includes gynecologists. Surgical treatment of CP is advocated. Diaphragmatic defects are observed in 29 to 66% of CP [25], suggesting that the trans-diaphragmatic passage of air coming from the uterus and tubes during pneumoperitoneum is probably the most frequent mechanism of pneumothorax in these patients [26]. Other findings found in CP might be pleural and/or parenchymal nodules suggestive of endometriosis. Gynecologists must evaluate the indication for laparoscopy (that can be carried out simultaneously or in a staged manner with VATS) and plan a perioperative hormonal suppression [22,27].

As previously stated, preventing recurrence is the other endpoint of surgical treatment of SP. There is not a clear and established definition of postoperative recurrence of pneumothorax. Woo and colleagues attempted in their study to suggest a new classification system for the postoperative recurrence of SP [4] and tried to identify risk factors that could be related to a second recurrence. Following other reports, authors concluded that a pneumothorax occurring within 30 days from surgery should not be considered a true recurrence but part of the healing process [4,28,29]. They demonstrated that patients with postoperative recurrence within 30 days showed a better prognosis than patients with late recurrent episodes.

Several interesting subjects have been debated in this Special Issue, presenting an updated picture of diagnosis, treatment, and recurrence of SP. Few questions remain open such as the size of the chest tube, medical or surgical thoracoscopy, and optimal treatment of recurrence.

As one of the Guest Editors, I would like to thank all the authors for their high-quality contributions, the reviewers for their professional help, and the *JCM* team for their support.
